# Feasibility of Home-Based Pulmonary Rehabilitation of Pediatric Patients with Chronic Respiratory Diseases

**DOI:** 10.3390/children11050534

**Published:** 2024-04-29

**Authors:** Da Yeong Kim, Young Hoon Mo, Kun Woo Kim, Sae Mi Hong, Arum Park, Baek Hee Jang, Seung Hak Lee, Joon Hee Lee, Jisun Yoon, Jinho Yu, Eun Jae Ko

**Affiliations:** 1 Department of Rehabilitation Medicine, Asan Medical Center, University of Ulsan College of Medicine, Seoul 05505, Republic of Korea; d200627@amc.seoul.kr (D.Y.K.); fevernova31@amc.seoul.kr (B.H.J.); d190296@amc.seoul.kr (S.H.L.); d200702@amc.seoul.kr (J.H.L.); 2 Department of Rehabilitation Medicine, Asan Medical Center, Seoul 05505, Republic of Koreara01637@amc.seoul.kr (K.W.K.); slpsaemi@amc.seoul.kr (S.M.H.); 3 Asan Institute for Life Sciences, Asan Medical Center, Seoul 05505, Republic of Korea; ra00387@amc.seoul.kr; 4 Department of Pediatrics, Chung-Ang University Gwangmyeong Hospital, College of Medicine, Chung-Ang University, Gwangmyeong 14353, Republic of Korea; jsyoon0507@cauhs.or.kr; 5 Department of Pediatrics, Asan Medical Center, University of Ulsan College of Medicine, Seoul 05505, Republic of Korea

**Keywords:** pulmonary rehabilitation, chronic respiratory disease, children, home-based

## Abstract

Background and objective: Chronic respiratory diseases in children deteriorate their daily life due to dyspnea and reduced lung function. We aimed to evaluate the feasibility of home-based pulmonary rehabilitation in pediatric chronic respiratory diseases. Methods: This prospective, single-arm, cohort study included children with chronic lung disease. They were instructed to perform home-based pulmonary rehabilitation 30 min/session, three sessions/week for three months. Pulmonary function test (PFT) using spirometry, respiratory muscle strength (RMT), cardiopulmonary exercise test (CPET), 6 min walk test (6MWT), dyspnea questionnaires, speech evaluation, and pediatric quality of life inventory (PedsQL) were assessed pre- and post-pulmonary rehabilitation. Compliance and satisfaction of the program were also evaluated. Results: Twenty children (mean age: 11.2 ± 3.1 years) with chronic respiratory diseases without cardiopulmonary instability participated. The overall compliance was 71.1% with no related adverse events. After pulmonary rehabilitation, forced expiratory volume in one second (FEV_1_), peak expiratory flow (PEF), RMT, 6MWT, dyspnea questionnaire, speech rate, and PedsQL (child) significantly improved (*p* < 0.05), particularly better in the FEV_1_ < 60% group than in the FEV_1_ ≥ 60% group and in the high-compliance group (compliance ≥ 50%) than in the low-compliance group (compliance < 50%). Conclusions: Home-based pulmonary rehabilitation for children with chronic lung disease was feasible with high compliance and effective in terms of objective functions, subjective dyspnea symptom, and quality of life.

## 1. Introduction

Chronic respiratory diseases in children include asthma, bronchiolitis obliterans (BO), and bronchopulmonary dysplasia (BPD) [1,2]. These conditions lead to dyspnea and diminish the quality of life of pediatric patients. The decreased pulmonary function in these patients gradually worsens as they age, which can subsequently result in chronic obstructive pulmonary disease (COPD) in adulthood [3,4]. Duan et al. reported that the risk of developing COPD is 3.45 times higher in children with asthma than in children without asthma [4]. In addition, BPD patients also experience immature lung development from preterm to infancy, which ultimately results in failure to obtain normal maximal forced expiratory volume in one second (FEV_1_) in adulthood. Gradual decrement of FEV_1_ after early adulthood of BPD patients is associated with decreased survival rates [5].

To enhance their quality of life, children with asthma should perform aerobic, strength exercises, and respiratory muscle training (RMT) to improve pulmonary function and enhance exercise capacity in children with asthma. Francois et al. conducted a 6-week aerobic exercise intervention on 16 male children with mild to moderate asthma and reported an increase in maximal oxygen uptake (VO_2_max) despite no changes in pulmonary function tests (PFT) [6]. In other studies, aerobic exercise did not significantly improve lung function, but it did enhance cardiorespiratory functions such as VO_2_max and maximal heart rate (HRmax) [7] as well as the quality of life [8,9]. The application of RMT has been explored in patients with chronic lung disease, including pediatric cases, and improvements were reported in maximal inspiratory pressure (MIP) and maximal expiratory pressure (MEP) in children with chronic lung disease [10].

Although there is limited research on the effects of resistance training in pediatric lung disease patients, some studies have investigated its effects on adult patients with COPD and reported improvements in the quality of life and the 6-minute walk test (6MWT) following resistance training [11]. Mortality could be predicted by the cross-sectional area of the midthigh muscle in COPD patients, which underscores the importance of muscle mass in individuals with chronic lung disease, further suggesting the need for strengthening exercises [12]. Pulmonary rehabilitation, including a combination of aerobic and strengthening exercise, improves respiratory muscle strength and enhances exercise capacity.

Unlike asthma, BO and BPD have been associated with the need for pulmonary rehabilitation to enhance a comprehensive therapy for children with these conditions. Moreover, it has been challenging for children to perform aerobic exercises, strengthening exercises, and RMT at home, resulting in effective implementation of rehabilitation therapy. This study aims to implement a well-designed home-based rehabilitation program for children with various chronic respiratory conditions, including BO and BPD, and to evaluate the feasibility of the home-based-pulmonary rehabilitation and further its effectiveness.

## 2. Methods

The study was approved by the Institutional Review Board (IRB) of the Asan Medical Center (IRB No. 2022-0328). The study was registered at Clinical Research Information Service (reference number: KCT0007586)

### 2.1. Participants

This single-center, prospective, single-arm, cohort study included 22 pediatric patients aged between 7 and 18 years who visited the outpatient clinic of Asan Medical Center for chronic respiratory diseases including BO, BPD, and other diseases. Those who had parental consent were included. Those who were inapplicable home-based pulmonary rehabilitation due to cardiopulmonary instability were excluded.

### 2.2. Home-Based Pulmonary Rehabilitation Program

The home-based pulmonary rehabilitation program was a 3-month program with >30 min per session, three sessions per week. It is composed of aerobic, strengthening exercises, and RMT. Patients were encouraged to follow a video program (https://www.youtube.com/watch?v=MTpW5UhEsHw assessed on 14 September 2022) for aerobic and strengthening exercise. The video program consists of 5 min of warm-up exercises, 25 min of aerobic exercises, 25 min of strengthening exercises, and 5 min of cool-down exercises.

Before performing the exercise at home, the patients received three rounds of orientation on the appropriate exercise techniques for these activities.

The intensity of aerobic and strengthening exercises was determined based on the results of the CPET or 6MWT [13]. For the patients who could perform CPET, the VO_2_max was converted to metabolic equivalents (METs) and initial exercise recommendations were made based on percent intensity (%Intensity) of METs according to their FEV_1_ severity (50% intensity for FEV_1_ < 60% and 70% intensity FEV1 > 60%, respectively) as defined in [14]:

Target METs = %Intensity × measured VO_2_ max(mL/kg/min) ÷ 3.5

The target heart rate was determined based on the HRmax during CPET by the Karvonen method [15]. The patients were encouraged to exercise once the target heart rate was achieved, and smartwatches (Miband 4, Xiaomi, Nanshan, Shenzhen, China) were provided to monitor their heart rates during exercise sessions at home. For children who could not perform CPET, the exercise intensity was calculated based on the maximum walking distance in 6MWT (6MWD). The initial exercise speed was set at 70% and 50% of their maximum for the FEV_1_ ≥ 60% group and the FEV_1_ < 60% group, respectively, as shown in the equation below [14]:

Target walking velocity (km/h) = % Intensity × 6MWD × 10 ÷ 1000 km/h;

they were gradually encouraged to walk at a faster pace.

The RMT utilized threshold load valves (IMT/PEP; Philips Respironics, Murrysville, PA, USA) daily. The daily inspiratory and expiratory muscle trainings each consisted of three sets of 10 min of ventilation and three sets of 15 exhalations, respectively, using the valve, both with a load of 30–50% of the MIP and MEP, respectively [16].

During the program, children maintained their usual sports and activities at school and in daily life.

### 2.3. Measurements

Pulmonary function test (PFT), MIP, MEP, CPET, and 6MWT were assessed before and after home-based pulmonary rehabilitation. The severity of dyspnea was measured using the mMRC, Pediatric Dyspnea Scale (PDS), and oxygen cost diagram (OCD). The quality of life was evaluated using the Pediatric Quality of Life inventory (PedsQL) for both parents and children. After the completion of the pulmonary rehabilitation, compliance and satisfaction of the intervention was assessed.

#### 2.3.1. Pulmonary Function Test (PFT)

PFT (V6200 Autobox Body Plethysmograph, SensorMedics, Yorba Linda, CA, USA) was conducted by spirometry to measure forced vital capacity (FVC), FEV_1_, FEV_1_/FVC ratio, peak inspiratory flow (PIF), and peak expiratory flow (PEF) according to American Thoracic Society (ATS) guideline [17].

#### 2.3.2. Respiratory Muscle Strength

MIP and MEP were measured using a desktop spirometer (Pony FX, Cosmed, Rome, Italy), which indicates respiratory muscle strength. MIP was measured by initiating a maximal inspiratory effort from residual volume and maintaining it for at least 1 s. MEP, however, was measured by initiating a maximal expiratory effort from total lung capacity and maintaining it for at least 1 s [10].

#### 2.3.3. Cardiopulmonary Exercise Test (CPET)

The CPET was conducted in adherence to established watt ramp protocols using a cycle ergometer (VIAsprint 150P; Carefusion, San Diego, CA, USA) under the supervision of a physician. The test was terminated in case of severe desaturation, ischemic ECG change, and systolic BP drop of >20 mmHg from the peak during the test [18]. Due to the required minimal distance between the pedal and saddle of the ergometer, some patients with shorter leg lengths were unable to perform CPET.

The CPET consisted of four stages [19]. The first was a resting stage, which is a 2 min rest period prior to the test. The second was a warm-up stage in which baseline data such as ECG, heart rate, oxygen saturation, blood pressure, and expired gas analysis were collected, and the patients performed unloaded pedaling at 30 rpm for 90 s. The third was an exercise stage in which the patients engaged in maximal exercise by pedaling at 60 rpm while gradually increasing the workload by 4–6 watts per minute. Borg dyspnea scale ratings were recorded throughout the exercise stage every 2 min. The test was stopped either at the participant’s request or in case of any abnormal medical findings. If a patient requested to discontinue the test, they proceeded to the recovery stage. The fourth was a recovery stage in which the patients performed pedaling without loading at 30–40 rpm for 2 min, and physiological data were collected until the patient stabilized. The maximal value of VO_2_ (VO_2_max), maximal heart rate (HRmax), saturation during exercise, and respiratory quotient were achieved from the test.

#### 2.3.4. 6 Min Walk Test (6MWT)

The 6MWT was performed in adherence to the guidelines set by the ATS [20]. Prior to the test, measurements of the Borg scale, heart rate, and oxygen saturation were obtained. These same measurements were also obtained immediately after the completion of the test.

#### 2.3.5. Severity of Dyspnea

Perceived dyspnea of participants was measured by Modified Medical Research Council (mMRC), Pediatric Dyspnea Scale (PDS), and oxygen cost diagram (OCD) in pre- and post- intervention. The mMRC is a 5-point scale based on the dyspnea during daily activities (0: least and 4: most difficult) [21]. The mMRC scale was performed by asking the level of their breathing difficulty in some activities. The PDS is a 7-level stratified image scale for how much trouble patients are having breathing. Level 1 indicates “No trouble at all” breathing, and level 7 is “Very much trouble”. The patients were requested to select on a diagram the level of breathing difficulty [22]. The OCD is a self-evaluation tool that assesses the dyspnea by selecting what activities are limited by shortness of breath [23]. This is a 10 cm vertical line divided into common daily activities according to their oxygen cost. At the bottom of the line is sleep, which indicates the smallest oxygen demand. At the top of the line is walking fast uphill as it requires the most energy. In this study, the patients were requested to mark on the point of line their perceived difficulty of dyspnea during the illustrated activities.

#### 2.3.6. Quality of Life

The quality of life deteriorated by dyspnea was evaluated using PedsQL^TM^ 4.0 (PedsQL) before and after intervention. PedsQL is an assessment of health-related quality of life for children with various health conditions. The PedsQL has 23 items related to physical functioning, emotional functioning, social functioning, and school functioning [24]. Each item is scored on a 5-point scale (0 = never, 1 = almost never, 2 = sometimes, 3 = often, 4 = almost always has a problem in the past 1 month), and a larger score means worse symptoms.

#### 2.3.7. Speech Evaluation

Speech evaluation, including maximal phonation time (MPT), speech rate, and speech handicap index, were conducted only in the patients who agreed to it. MPT is a task that involves prolonging the vowel “ah” as much as possible after taking a deep breath, and the length of phonation is measured in seconds. MPT can provide information about respiratory and vocal system issues in individuals with speech disorders [25]. The speech rate was applied using a Korean version paragraph to measure the time taken to read the entire paragraph [26]. The Korean version of the speech handicap index is also used to evaluate a patient’s perceived speech dysfunction in daily life. The speech handicap index consists of three sets: speech functional (14 items), psychosocial functional (14 items), and other parts (2 items). For each item, 0 point represents “not at all”, while 4 points is “always”. The total score ranges from 0 to 120, with higher scores indicating a speech dysfunction [27].

#### 2.3.8. Feasibility Measure

Compliance with home-based pulmonary rehabilitation was measured by counting the number of exercise sessions attended out of a total of 36 sessions, based on the criteria of three sessions per week for 3 months. An online self-reporting exercise poll was conducted, and the frequency was recorded.

To evaluate satisfaction, the self-report questionnaire (Table S1) was conducted after the intervention. It consists of two elements: a questionnaire about satisfaction with the video rehabilitation program (5 questions) and another questionnaire about the changes in daily life after the program (4 questions). It is composed of a 5-point scale (1 = extremely satisfied, 2 = very satisfied, 3 = sometimes satisfied, 4 = almost never satisfied, and 5 = never satisfied), giving a total score of 9–45 points. A lower score means higher satisfaction about the rehabilitation program.

#### 2.3.9. Other Clinical Variables

Age, gender, weight, height, diagnosis, and current medications were investigated in all patients. To determine the exacerbation of the disease during the study, emergency room visits and hospital admissions were also assessed.

### 2.4. Statistical Analysis

Continuous variables were expressed as mean values and standard deviations. Paired comparisons for pre- and post-pulmonary rehabilitation values were analyzed using the paired T-test for normal distribution data and Wilcoxon rank-sum test for non-normal distribution data. Subgroup analysis based on compliance (compliance ≥ 50% and compliance < 50%) and severity (FEV_1_ ≥ 60% and FEV_1_ < 60%) was performed using the Mann–Whitney U-test. SPSS version 25 (IBM Co., Armonk, NY, USA) was used for statistical analysis, and *p* < 0.05 was considered statistically significant.

## 3. Results

### 3.1. Baseline Characteristics

In this study, two of the 22 children waived participation, due to living a long distance away from the hospital and having academic issues. The demographic and baseline characteristics of participants are shown in Table 1. Among the 20 included patients, 10 were male (50%), and the mean age was 11.2 ± 3.1 (7–17) years. The diagnosis of children consisted of BO (*n* = 12), BPD (*n* = 5), asthma (*n* = 1), status post lung transplantation (*n* = 1), and postinfectious lung after acute respiratory distress syndrome (ARDS) (*n* = 1). The time since diagnosis and treatment was 7.7 ± 3.7 years. Based on the severity of asthma according to FEV_1_, 3 (15%) patients had an FEV_1_ ≥ 60%, while 17 (85%) patients had an FEV_1_ < 60%, which means that 85% of the patients had severe lung function [28]. PFT revealed that an obstructive pattern was the most common type (75%), followed by mixed type (15%), and restrictive type (10%). Twelve participants were using short-acting beta agonists (SABAs) on an as-needed (prn) basis due to controlled symptoms. Three were on daily low-dose inhaled cortical steroid (ICS)/long-acting muscarinic antagonist (LAMA), and three were using daily ICS alone. One participant, who had a lung transplant, was using a combination of LAMA, oral steroids, ICS, and leukotriene inhibitors. Except for one individual, none of these patients experienced any changes in medication before and after pulmonary rehabilitation.

### 3.2. Compliance and Satisfaction of the Program

There was one event of emergency room visit by one patient due to dyspnea, which was not caused by the exercise, but by experiencing a worsening of a pre-existing condition due to COVID-19, two months after starting the rehabilitation program. This individual, who had been taking ICS and leukotriene inhibitors, stopped taking ICS and started high-dose steroids with a gradual taper. Therefore, this patient was not dropped out during the intervention. 

Compliance of the home-based pulmonary rehabilitation program was 71.1%, and satisfaction of program was 17.1 points (Table 2).

### 3.3. Effects of Home-Based Pulmonary Rehabilitation

Table 3 compares the evaluation of variables between pre- and post-pulmonary rehabilitation. After pulmonary rehabilitation, there were significant differences in FVC (1.85 ± 0.58–2.01 ± 0.60 L/s, *p* = 0.001) and FEV_1_ (1.07 ± 0.36–1.17 ± 0.38 L/s, *p* < 0.001); however, it did not meet the minimal clinically important difference (MCID) of asthma which is above 10% [29]. There were also improvements in PIF (1.89 ± 0.77–2.42 ± 1.03 L/s, *p* = 0.045) and PEF (2.60 ± 0.94-2.82 ± 0.79 L/s, *p* = 0.020). In respiratory muscle strength, improvements were observed in MIP (34.15 ± 18.07–47.20 ± 22.26 cmH_2_O, *p* < 0.001) and MEP (36.95 ± 16.02–47.40 ± 15.52 cmH_2_O, *p* < 0.001). In 12 patients who performed CPET, the VO_2_max and METs of CPET improved, but they were not statistically significant. The 6MWD also improved from 457.75 ± 117.48 to 515.73 ± 101.83 m (*p* = 0.003). Dyspnea questionnaire of mMRC (*p* = 0.038), OCD (*p* = 0.004), and PedsQL (child) (*p* = 0.004) also improved. Speech evaluation was conducted on eight patients, with a significant difference in speech rate (104.75 ± 31.11–89.50 ± 20.73 s, *p* = 0.021).

### 3.4. Effect of Home-Based Pulmonary Rehabilitation According to Compliance

Compliance was divided based on a threshold of 50% (compliance ≥ 50%, *n* =15; compliance < 50%, *n* = 5) (Table 4). In the compliance ≥ 50% group, significant differences were observed in FVC (1.78 ± 0.53 L/s–1.96 ± 0.55 L/s, *p* = 0.005), FEV_1_ (1.07 ± 0.31 L/s–1.19 ± 0.55 L/s, *p* = 0.001), PIF (1.72 ± 0.64 L/s–2.47 ± 0.94 L/s, *p* = 0.001), PEF (2.52 ± 0.97 L/s–2.86 ± 0.80 L/s, *p* = 0.014), MIP (33.27 ± 19.12 cmH_2_O–45.87 ± 23.66 cmH_2_O, *p* < 0.001), MEP (37.00 ± 17.35 cmH_2_O–47.13 ± 16.17 cmH_2_O, *p* = 0.002), 6MWD (455.70 ± 135.62 m–519.53 ± 108.77 m, *p* = 0.008), mMRC (1.33 ± 1.35–0.80 ± 1.21, *p* = 0.038), OCD (69.97 ± 17.77–77.67 ± 13.21, *p* = 0.008), and PedsQL (child) (22.00 ± 18.03–12.73 ± 11.74, *p* = 0.003). However, in the compliance < 50% group, only MEP (36.80 ± 12.87 cmH_2_O–48.20 ± 15.09 cmH_2_O, *p* = 0.043) showed significant improvement. The comparison between the two groups was determined by the difference between pre- and post-rehabilitation values. Significant differences were observed in FVC (*p* = 0.026), FEV_1_ (*p* = 0.011), PIF (*p* = 0.040), and PEF (*p* = 0.029), with greater improvements in the compliance ≥ 50% group than in the compliance < 50% group.

### 3.5. Effect of Home-Based Pulmonary Rehabilitation according to the Severity of the Disease

Severity was divided into two groups based on the cutoff of 60% (Table 5). The FEV_1_ ≥ 60% group consisted of three patients, while the FEV_1_ < 60% group consisted of 17 patients. In the FEV_1_ < 60% group, significant improvements were observed in FVC (1.91 ± 0.47 L/s–2.07 ± 0.51 L/s, *p* = 0.028), FEV_1_ (1.05 ± 0.30 L/s–1.17 ± 0.33 L/s, *p* = 0.001), PIF (1.86 ± 0.74 L/s–2.52 ± 1.04 L/s, *p* = 0.010), MIP (35.94 ± 18.78 cmH_2_O–50.65 ± 21.83 cmH_2_O, *p* < 0.001), MEP (38.00 ± 16.83 cmH_2_O–48.47 ± 16.64 cmH_2_O, *p* = 0.001), mMRC (1.35 ± 1.27–0.88 ± 1.17, *p* = 0.038), OCD (68.79 ± 16.48–75.59 ± 13.10, *p* = 0.008), and PedsQL (child) (24.53 ± 17.67–16.71 ± 14.74, *p* = 0.006). However, in the FEV_1_ ≥ 60% group, no statistically significant improvements were observed. Likewise, there were no statistically significant differences observed between the groups.

## 4. Discussion

This study demonstrated significant improvements in lung function, aerobic function, speech function, subjective dyspnea symptom, and quality of life in children with various chronic lung diseases after 3 months of home-based pulmonary rehabilitation. These improvements were more marked in the severe lung function group and the high compliance group [14]. Furthermore, this home-based pulmonary rehabilitation program showed high compliance rates and did not lead to any severe adverse events.

The average compliance of our program was 71.1%, which was higher than that in other studies of home-based pulmonary rehabilitation [30,31,32]. In the present study, compliance was achieved by >50% in 15 patients, and >80% in 10 patients. Compliance is very important in pulmonary rehabilitation to reach the optimal effectiveness, especially in patients with obstructive diseases [31,33]. It is expected that a high compliance in this study would also have resulted in good outcomes for the children with chronic lung diseases. Furthermore, the dropout rate in this study was 9.1%, which was lower than that in other home-based exercise programs [28,34]. Although most of the patients had severe or moderate pulmonary disease, there was no adverse event as a reason for dropout during pulmonary rehabilitation. This indicates the safety and the good tolerance of the program used in this study.

The satisfaction survey showed an average of 17.1 ± 5.0 points, indicating that the level of satisfaction was relatively high. The patients reported that the pulmonary rehabilitation using a video program was convenient, interesting, and helpful and for the images and sound, they were very to extremely satisfied. After the intervention, the patients felt improvement in their dyspnea and more interest in respiratory health. However, three months of exercise program could not change the exercise habits of the patients, thus requiring more efforts to make routine exercise their habit.

The results of the present study demonstrate the effectiveness of the home-based pulmonary rehabilitation in children with various chronic respiratory diseases. RMT contributes to the improvements in MIP and MEP and the prevention of the bronchiole obstruction with the increase in the bronchus airway, resulting in the improvement in lung capacity such as FVC, and FEV_1_ [35]. An increase in pulmonary function and limb muscle power improves exercise tolerance, as indicated by 6MWD. When considering exercise tolerance, we would have expected to see a significant improvement in CPET’s VO_2_max, but this was not statistically significant. This lack of statistical significance could be because we were able to conduct tests on only 12 out of 20 participants, leading to insufficient data to confirm significant results. Furthermore, pulmonary rehabilitation comprising stretching, aerobic, and strengthening exercises can coordinate the diaphragm and abdominal muscles and amplify the range of motion of the thorax [35]. Pulmonary rehabilitation can improve dyspnea symptoms and improve the quality of life, as indicated by the results of the dyspnea questionnaire, and PedsQL (child) also improved statistically significantly (*p* < 0.05) after the intervention. 

A previous study showed the effectiveness of 6 weeks of RMT after MIP, MEP, and 6MWT [36]; however, this study showed improvements in FVC, FEV_1_, PIF MIP, MEP, and 6MWT after a combination of aerobic exercise, strengthening exercise, and RMT. The difference in the results might be due to the complexity of the program (RMT vs. RMT, aerobic and strengthening), or the duration of the intervention (6 weeks vs. 3 months) between the studies.

Regarding the effects of compliance, the patients with good compliance showed improvements in PEF, as indicated by lower resistance of the bronchus, confirming the effectiveness of the pulmonary rehabilitation. Conversely, those with poor compliance exhibited decreased FVC, PEF, and PIF, suggesting the importance of the compliance of the intervention. The differences between groups based on compliance were statistically significant only for PIF and PEF, but not for the other parameters, possibly because of the small number of patients in the group with low compliance (*n* = 5).

When the effect of the program was investigated according to the severity of the pulmonary disease, significant improvements were observed in most parameters, including PFT (except for FEV_1_/FVC), RMT, 6MWD, dyspnea questionnaire, and QoL in the FEV_1_ < 60% group. The FEV_1_ ≥ 60% group showed no significant results, and this may be due to the limited sample size of three individuals. Furthermore, there was no significant difference observed between the two groups, indicating that the program was effective in both the FEV_1_ < 60% group and FEV_1_ ≥ 60% group. Another previous study investigated a group of patients with mild pulmonary disease and demonstrated that home-based interventions are effective [8]. Therefore, our findings showed similar results since we recruited a larger number of participants for the mild and moderate groups, thereby demonstrating the effectiveness of the intervention in the FEV_1_ < 60% group. However, since there are fewer patients in a group (*n* = 5), we must be careful in interpreting Table 4 and Table 5.

Speech rate is used to evaluate the time taken to speak the same paragraph composed of 210 syllables. Improved pulmonary function may result in increased number of syllables per time when talking, by reduction in pauses for breathing during talking. In this study, only eight patients underwent speech evaluation because the evaluation was started in the middle of the study. These patients showed improvement in MPT, speech rate, and speech handicap index, but only speech rate was statistically significant, probably due to the small number of patients. Considering this result, we can also assume that the patients are less breathless after the intervention [37].

To the best of our knowledge, this is the first study that applied home-based pulmonary rehabilitation to children with BO and BPD and identified improved both objective measurements and subjective dyspnea and QoL. Furthermore, we provided home-based pulmonary rehabilitation that showed relatively high compliance and satisfaction. Since hospital-based pulmonary rehabilitation is difficult to complete in consideration of patient compliance, it is important to prove the effectiveness of the home-based pulmonary rehabilitation.

There are some limitations to this study. It was a single-arm study without a control group, the total number of patients and the number of children with mild to moderate disease severity was small, and the diseases of the patients were diverse. Although the participants are pediatric, the wide range of ages and varying time since diagnosis are also limitations. Larger-scale studies are needed in the future to provide evidence regarding the feasibility of rehabilitation in a larger number of patients, including those in the FEV_1_ ≥ 60% group. Such studies would help further explore the potential of rehabilitation in these populations. Furthermore, we did not restrict the usual sports and activities of the children, and this might have affected the results. In addition, to confirm whether the improved lung function through pulmonary rehabilitation will persist and help normal lung growth of the children, further study with long-term follow-up is needed. To explore the mechanism of the pulmonary rehabilitation in these patients, further molecular study is also needed.

## 5. Conclusions

A home-based pulmonary rehabilitation program is feasible with high compliance, and is safe for children with chronic respiratory diseases. After pulmonary rehabilitation, significant improvements were observed in subjective measurements such as dyspnea scale and QoL, as well as the objective measurements including PFT, respiratory muscle strength, and 6MWT, as demonstrated by the FEV_1_ < 60% group and high-compliance group. To establish the effects of a home-based pulmonary rehabilitation on other types of chronic lung disease, further studies should include a larger population with diverse severity.

## Figures and Tables

**Table 1:** Baseline characteristics of pediatric patients with chronic lung diseases.

Characteristics	
Age, mean (range)	11.2 ± 3.1 (7–17)
Sex (male/female)	10 (50%)/10 (50%)
Height (cm)	144.1 ± 16.2
Weight (kg)	37.5 ± 14.2
**Diagnosis**	
Bronchiolitis obliterans	12 (60%)
Bronchopulmonary dysplasia	5 (25%)
ETC	3 (15%)
Time since diagnosis (years)	7.7 ± 3.7
**Severity**	
FEV_1_ ≥ 80%	0 (0%)
80% > FEV_1_ ≥ 60%	3 (15%)
FEV_1_ < 60%	17 (85%)
**Type**	
Obstructive	15 (75%)
Restrictive	2 (10%)
Mixed	3 (15%)
**Maintenance therapy**	
Prn SABA	12 (60%)
Daily ICS	3 (15%)
Daily ICS/LAMA	3 (15%)
Other (mixed therapy)	2 (10%)

Results are expressed as mean ± SD (range) or number (%). ETC included lung transplantation status, asthma, and status of postacute pulmonary lung disease. FEV_1_: forced expiratory volume in the first second. SABA: short-acting beta agonist. ICS: inhaled corticosteroid. LAMA: long-acting muscarinic antagonist. Prn: as needed.

**Table 2:** Results of the questionnaires after home-based pulmonary rehabilitation program.

**Total**	17.1 ± 5.0
**Video program satisfaction**	
Convenience	1.8 ± 0.7
Interesting	2.0 ± 0.9
Image and design	2.0 ± 0.8
Sound and song	1.8 ± 0.8
Helpfulness	1.8 ± 0.7
**Changes of daily life after participating in the intervention**	
Feel my breathing improving	1.9 ± 0.6
More interested in respiratory health	1.7 ± 0.8
Do more walking exercises	2.3 ± 0.8
Do more breathing exercises	2.1 ± 0.5

Results are expressed as mean ± SD (range).

**Table 3:** Effect of home-based pulmonary rehabilitation.

	Pre-Rehab	Post-Rehab	*p*-Value
**Pulmonary function test**			
FVC (L/s)	1.85 ± 0.58 (70.50 ± 13.27%)	2.01 ± 0.60 (75.80 ± 12.49%)	0.001
FEV_1_ (L/s)	1.07 ± 0.36 (45.90 ± 14.04%)	1.17 ± 0.38 (50.20 ± 14.39%)	<0.001
FEV_1_/FVC (%)	61.00 ± 17.67	60.15 ± 15.93	0.513
PIF (L/s)	1.89 ± 0.77	2.42 ± 1.03	0.045
PEF (L/s)	2.60 ± 0.94	2.82 ± 0.79	0.020
**Pulmonary muscle strength**			
MIP (cmH_2_O)	34.15 ± 18.07	47.20 ± 22.26	<0.001
MEP (cmH_2_O)	36.95 ± 16.02	47.40 ± 15.52	<0.001
**CPET**			
VO_2_max	19.85 ± 5.34	22.09 ± 8.31	0.134
METs	5.71 ± 1.49	6.31 ± 2.38	0.160
RQ	1.14 ± 0.12	1.24 ± 0.14	0.108
**6 min walking test**			
6MWD (m)	457.75 ± 117.48	515.731 ± 101.83	0.003
6MWT Borg scale	11.20 ± 3.14	12.20 ± 3.83	0.176
**Dyspnea Questionnaire**			
mMRC	1.15 ± 1.27	0.75 ± 1.12	0.038
PDS	2.00 ± 1.08	1.65 ± 0.99	0.109
OCD	74.54 ± 11.79	77.25 ± 12.82	0.004
**QoL**			
PedsQL (child)	23.45 ± 16.46	16.35 ± 13.59	0.004
PedsQL (parent)	25.10 ± 19.18	22.60 ± 18.80	0.456
**Speech evaluation**			
Maximal phonation time (s)	7.68 ± 2.28	8.91 ± 1.91	0.069
Speech rate (s)	104.75 ± 31.11	89.50 ± 20.73	0.021
Speech handicap index	14.25 ± 13.56	12.38 ± 12.87	0.266

Results are expressed as mean ± SD (range). *p* < 0.05 (paired *t*-test, Wilcoxon rank-sum test). Pre-rehab: pre-rehabilitation; Post-rehab: post-rehabilitation; FVC: forced volume vital capacity; FEV_1_: forced expiratory volume in the first second; PIF: peak inspiratory flow; PEF: peak expiratory flow; MIP: maximal inspiratory pressure; MEP: maximal expiratory pressure; CPET: cardiopulmonary exercise test; VO_2_max: maximal oxygen consumption; MET: metabolic equivalent of task; RQ: respiratory quotient; 6MWD: 6 min walking distance; 6MWT: 6 min walking test; mMRC: Modified Medical Research Council; PDS: Pediatric Dyspnea scale; OCD: oxygen cost diagram; QoL: quality of life; PedsQL: Pediatric Quality of Life Inventory.

**Table 4:** Effect of home-based pulmonary rehabilitation according to the compliance.

	Compliance ≥ 50% (*n* = 15)			Compliance < 50% (*n* = 5)			
	Pre-Rehab	Post-Rehab	*p*-Value	Pre-Rehab	Post-Rehab	*p*-Value	*p*-Value between the Group Differences
**Pulmonary function test**							
FVC (L/s)	1.78 ± 0.53 (67.40 ± 12.40%)	1.96 ± 0.55 (75.00 ± 13.28%)	0.005	2.05 ± 0.76 (79. 80 ± 12.40%)	2.13 ± 0.77 (78.20 ± 10.66%)	0.416	0.026
FEV_1_ (L/s)	1.07 ± 0.31 (45.40 ± 13.55%)	1.19 ± 0.55 (51.07 ± 14.08%)	0.001	1.08 ± 0.53 (47.40 ± 17.02%)	1.13 ± 0.52 (47.60 ± 16.68%)	0.785	0.011
FEV_1_/FVC (%)	62.93 ± 16.29	61.47 ± 14.56	0.391	55.20 ± 22.31	56.20 ± 20.91	0.269	0.379
PIF (L/s)	1.72 ± 0.64	2.47 ± 0.94	0.001	2.42 ± 0.95	2.26 ± 1.38	0.686	0.040
PEF (L/s)	2.52 ± 0.97	2.86 ± 0.80	0.014	2.83 ± 0.91	2.71 ± 0.85	0.345	0.029
**Pulmonary muscle strength**							
MIP (cmH_2_O)	33.27 ± 19.12	45.87 ± 23.66	<0.001	36.80 ± 12.87	51.20 ± 19.18	0.078	0.631
MEP (cmH_2_O)	37.00 ± 17.35	47.13 ± 16.17	0.002	36.80 ± 12.87	48.20 ± 15.09	0.043	>0.999
**CPET**							
VO_2_max	21.25 ± 4.20	23.69 ± 7.41	0.219	15.20 ± 7.05	17.30 ± 10.69	0.285	0.964
METs	6.10 ± 1.18	6.77 ± 2.13	0.239	4.40 ± 1.93	4.93 ± 3.04	0.593	0.964
RQ	1.14 ± 0.13	1.28 ± 0.14	0.077	1.14 ± 0.07	1.11 ± 0.07	0.109	0.115
**6 min walking test**							
6MWD (m)	455.70 ± 135.62	519.53 ± 108.77	0.008	463.90 ± 33.45	504.30 ± 87.36	0.225	0.513
6MWT Borg scale	11.53 ± 3.20	12.13 ± 3.52	0.454	10.20 ± 3.03	12.40 ± 5.13	0.197	0.329
**Dyspnea questionnaire**							
mMRC	1.33 ± 1.35	0.80 ± 1.21	0.038	0.60 ± 0.89	0.60 ± 0.89	>0.999	0.150
PDS	2.13 ± 1.19	1.67 ± 1.05	0.058	1.60 ± 0.55	1.60 ± 0.89	>0.999	0.425
OCD	69.97 ± 17.77	77.67 ± 13.21	0.008	74.00 ± 11.40	76.00 ± 12.94	0.157	0.214
**QoL**							
PedsQL (child)	22.00 ± 18.03	12.73 ± 11.74	0.003	27.80 ± 10.83	27.20 ± 14.10	0.854	0.072
PedsQL (parent)	25.73 ± 21.13	19.53 ± 19.48	0.116	23.20 ± 13.37	31.80 ± 14.46	0.345	0.066

Results are expressed as mean ± SD (range). *p* < 0.05 (paired *t*-test, Wilcoxon rank-sum test). *p*-value between group differences was evaluated by difference between pre- and post-rehabilitation values on each group. *p*-value < 0.05 (Mann–Whitney U-test). Pre-rehab: pre-rehabilitation; Post-rehab: post-rehabilitation; FVC: forced volume vital capacity; FEV_1_: forced expiratory volume in the first second; PIF: peak inspiratory flow; PEF: peak expiratory flow; MIP: maximal inspiratory pressure; MEP: maximal expiratory pressure; CPET: cardiopulmonary exercise test; VO_2_max: maximal oxygen consumption; MET: metabolic equivalent of task; RQ: respiratory quotient; 6MWD: 6 min walking distance; 6MWT: 6 min walking test; mMRC: Modified Medical Research Council; PDS: Pediatric Dyspnea Scale; OCD: oxygen cost diagram; QoL: quality of life; PedsQL: Pediatric Quality of Life Inventory; s: seconds.

**Table 5:** Effects of home-based pulmonary rehabilitation according to disease severity.

	FEV_1_ < 60% Group (*n* = 17)			FEV_1_ ≥ 60% Group (*n* = 3)			
	Pre-Rehab	Post-Rehab	*p*-Value	Pre-Rehab	Post-Rehab	*p*-Value	*p*-Value between the Group Differences
**Pulmonary function test**							
FVC (L/s)	1.91 ± 0.47 (70.24 ± 14.03%)	2.07 ± 0.51 (75.24 ± 13.04%)	0.028	1.50 ± 1.13 (72.00 ± 9.64%)	1.65 ± 1.06 (79.00 ± 10.15%)	0.285	0.750
FEV_1_(L/s)	1.05 ± 0.30 (42.59 ± 12.42%)	1.17 ± 0.33 (47.59 ± 13.96%)	0.001	1.15 ± 0.72 (64.67 ± 4.04%)	1.20 ± 0.69 (65.00 ± 4.58%)	0.785	0.110
FEV_1_/FVC (%)	57.29 ± 16.14	57.53 ± 15.59	0.799	82.00 ± 10.15	75.00 ± 8.72	0.285	0.311
PIF(L/s)	1.86 ± 0.74	2.52 ± 1.04	0.010	2.06 ± 1.09	1.83 ± 0.93	0.285	0.050
PEF (L/s)	2.58 ± 0.92	2.84 ± 0.77	0.051	2.69 ± 1.29	2.74 ± 1.13	>0.999	0.397
**Pulmonary muscle strength**							
MIP (cmH_2_O)	35.94 ± 18.78	50.65 ± 21.83	<0.001	24.00 ± 9.85	27.67 ± 14.84	0.593	0.100
MEP (cmH_2_O)	38.00 ± 16.83	48.47 ± 16.64	0.001	31.00 ± 10.54	41.33 ± 2.52	0.109	0.916
**CPET**							
VO_2_max	19.58 ± 5.48	21.44 ± 8.40	0.212	23.20 ± 0.00	29.20 ± 0.00	NA	0.765
METs	5.63 ± 1.53	6.13 ± 2.41	0.247	6.60 ± 0.00	8.30 ± 0.00	NA	0.765
RQ	1.14 ± 0.12	1.25 ± 0.14	0.087	1.15 ± 0.00	1.09 ± 0.00	NA	0.479
**6 min walking test**							
6MWD (m)	453.44 ± 127.22	505.91 ± 107.47	0.017	482.17 ± 24.17	571.33 ± 25.66	0.109	0.491
6MWT Borg scale	11.71 ± 3.04	12.47 ± 3.50	0.307	8.33 ± 2.31	10.67 ± 6.11	0.276	0.451
**Dyspnea Questionnaire**							
mMRC	1.35 ± 1.27	0.88 ± 1.17	0.038	0.00 ± 0.00	0.00 ± 0.00	>0.999	0.295
PDS	2.12 ± 1.11	1.77 ± 1.03	0.166	1.33 ± 0.58	1.00 ± 0.00	0.317	0.955
OCD	68.79 ± 16.48	75.59 ± 13.10	0.008	83.33 ± 7.61	86.67 ± 5.77	0.157	0.667
**QoL**							
PedsQL (child)	24.53 ± 17.67	16.71 ± 14.74	0.006	17.33 ± 3.06	14.33 ± 3.21	0.180	0.633
PedsQL (parent)	26.12 ± 20.08	23.47 ± 20.20	0.518	19.33 ± 14.67	17.67 ± 7.02	0.593	0.832

Results are expressed as mean ± SD (range). *p* < 0.05 (paired *t*-test, Wilcoxon rank-sum test). *p*-value between group differences was evaluated by difference between pre- and post-rehabilitation value on each group. *p*-value < 0.05 (Mann–Whitney U-test). Pre-rehab: pre-rehabilitation; Post-rehab: post-rehabilitation; FVC: forced volume vital capacity; FEV_1_: forced expiratory volume in the first second; PIF: peak inspiratory flow; PEF: peak expiratory flow; MIP: maximal inspiratory pressure; MEP: maximal expiratory pressure; CPET: cardiopulmonary exercise test; VO_2_max: maximal oxygen consumption; MET: metabolic equivalent of task; RQ: respiratory quotient; 6MWD: 6 min walking distance; 6MWT: 6 min walking test; mMRC: Modified Medical Research Council; PDS: Pediatric Dyspnea Scale; OCD: oxygen cost diagram; QoL: quality of life; PedsQL: pediatric quality of life inventory; s: seconds; NA: not available.

## Data Availability

The data that support the findings of this study are available on request from the corresponding author due to privacy and ethical reasons.

## Supplementary Materials

The following supporting information can be downloaded at: https://www.mdpi.com/article/10.3390/children11050534/s1, Table S1. Satisfaction questionnaires after home-based pulmonary rehabilitation program.
