# Supplementary material for: Feasibility of Home-Based Pulmonary Rehabilitation of Pediatric Patients with Chronic Respiratory Diseases
Source: Children (Basel). 2024 Apr 29;11(5):534. doi: 10.3390/children11050534 (PMC11119592; doi:10.3390/children11050534)
Supplement: Supplementary file 1 [file children-11-00534-s001.zip › children-2967176-supplementary.pdf]

Supplementary materials

Table S1. Satisfaction questionnaires after home-based pulmonary rehabilitation program

		extremely satisfied (1)	very satisfied (2)	sometimes satisfied (3)	almost never satisfied (4)	never satisfied (5)
**Ⅰ. Satisfaction with the video rehabilitation program**						
1	It is convenient to use.					
2	Interesting.					
3	I am satisfied with the image and design.					
4	I am satisfied with the song and sound.					
5	This program is needed for pediatric chronic lung disease patients.					
**Ⅱ. Changes in daily life after the program**						
1	I do more walking exercise after the program.					
2	I do more breathing exercises after the program.					
3	I became more interested in my respiratory health.					
4	I feel my breathing has improved.					
